# Evaluation of Direct and Indirect Pulp Capping With Biodentine in Vital Permanent Teeth With Deep Caries Lesions

**DOI:** 10.7759/cureus.39374

**Published:** 2023-05-23

**Authors:** Sofia Drouri, Hafsa El merini, Aly Sy, Mouna Jabri

**Affiliations:** 1 Department of Conservative Dentistry and Endodontics, Faculty of Dental Medicine, University Hassan II, Casablanca, MAR

**Keywords:** carious pulp exposure, vital pulp therapy, biodentine, indirect pulp capping, direct pulp capping

## Abstract

Introduction: Pulp capping is a procedural method of preserving pulp vitality following deep caries damage or accidental pulp exposure. Biodentine is a calcium silicate material that has been promoted for use in pulp capping among various clinical applications. This study evaluated the outcome of pulp capping with Biodentine following curettage of deep caries in the case series of permanent mature teeth.

Materials and methods: The study was carried out on 40 teeth with advanced caries treated by direct and indirect pulp capping using Biodentine, within a follow-up of six months. This was carried out at the Department of Conservative Dentistry-Endodontics of the CCTD Ibn Rochd-Casablanca.

Results: In this study, 43 teeth from 37 patients were treated with direct pulp capping and indirect pulp capping using Biodentine. The success rate of pulp capping was 90% at one month, 85% at three months, and 80% at six months.

Conclusion: The results of the studies conducted using Biodentine indicate that it is a suitable material for direct and indirect pulp capping, due to its bioactivity and ability to form a dentinal bridge.

## Introduction

Pulp capping is a minimal invasive vital therapy that preserves the pulp vitality of teeth with deep caries lesions or accidental pulp exposure [1,2]. The exposed dentin-pulp tissues are covered with a medication in contact with a pulp wound to promote dentin-pulp healing and/or obliteration of the exposed pulp by a neoformed dentin bridge [3]. Calcium hydroxide (CH) has long been a widely used material for pulp capping, forming a dentin bridge [4]. However, it has some disadvantages including pulp surface inflammation and necrosis after pulp capping, tunnel defects in the dentin bridge, porosity, and lack of adhesion which are secondary entry points for bacteria and their metabolites [3,5-7]. Thanks to their advantageous properties, such as bioactivity, water tightness, biocompatibility, and mechanical properties, calcium silicate materials, such as Biodentine^TM^ (Septodont Ltd., Saint Maur des Faussées, France) and mineral trioxide aggregate (MTA), have become the materials of choice for pulp capping. Biodentine is easy to use and has the advantage of not causing tooth discoloration compared to MTA [8,9]. Biodentine is a bioactive cement with mechanical properties similar to those of dentin that can be used as a substitute. When in direct contact with a vital pulp, it has been found to stimulate pulp cells and promote the formation of reparative dentin [10]. Indeed, Biodentine is an innovative dental material that stands apart from traditional Portland cement-based materials due to its lack of aluminic components and the enhanced physical properties achieved through its "Active Biosilicate Technology" process [10]. Indeed, the presence of finer particle size, use of zirconium oxide as a radiopacifier, purity of tricalcium silicate, absence of dicalcium silicate, and the addition of calcium chloride and hydrosoluble polymer improved the physical and biologic properties of Biodentine [11].

The objective of this study was to evaluate the outcomes of direct and indirect pulp capping with Biodentine in 40 cases after deep caries curettage, by conducting pulp sensitivity tests and analyzing radiographs at one, three, and six months.

## Materials and methods

Patients consulting the Department of Conservative Dentistry and Endodontics in Casablanca who met the following inclusion and exclusion criteria were included in the study (Table 1).

**Table 1:** Inclusion and exclusion criteria of the study

Inclusion criteria	Exclusion criteria
Patients between 15 and 55 years old	Inability to place the dental rubber dam
Teeth with deep and very deep caries without symptoms or irreversible pulpitis	Very large cavity needing endodontic post restoration
Less than 2 mm of carious exposure	Significant pulp bleeding > 2 minutes

**Figure 1:**
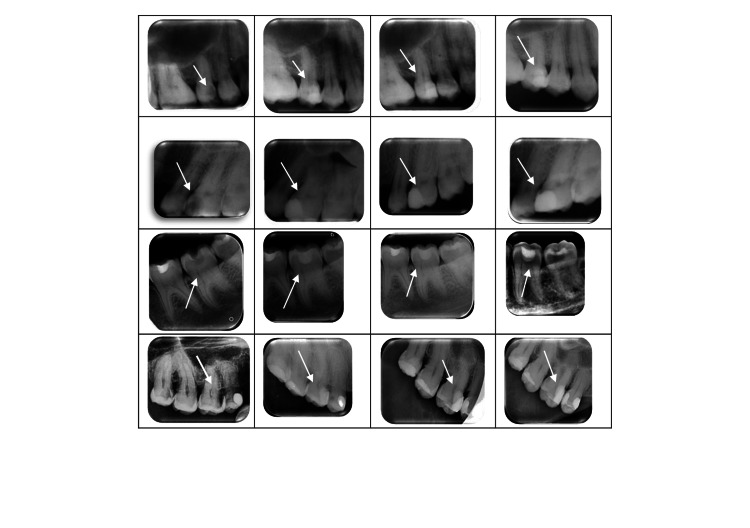
Examples of pre and postintervention radiographs. From left to right: preop radiograph, follow-up at one month, three months, and six months

Patients included in the study are provided with a detailed explanation of the procedure and the importance of preserving pulp vitality, and their informed and signed consent is obtained during the first consultation. A standard operating procedure, developed from several studies, is followed for all participants, starting with the diagnosis of pulp vitality, which is assessed with a cold thermal test using endo ice on the previously dried and isolated tooth in the vestibule [3,12-14]. Positivity or a short-lived sharp pain in response to the stimulus is compared with those of the contralateral vital teeth. Percussion and palpation are performed preoperatively and at one-month, three-month, and six-month follow-ups. Intra-oral radiography is also taken preoperatively. Local anesthesia is applied, followed by isolation of the tooth with a rubber dam. Caries removal is then conducted with a diamond ball bur and tungsten carbide ball bur mounted on a turbine and contra-angle, respectively, under constant irrigation. If any accidental exposure of the pulp occurs during curettage, immediate disinfection of the pulp wound with 2.5% sodium hypochlorite is undertaken. Hemostasis is then established using a cotton pellet impregnated with saline solution, followed by disinfection of the tooth with a cotton pellet impregnated with 2.5% sodium hypochlorite. The cavity is then rinsed with saline and gently dried with cotton pellets. The capping agent (Biodentine) is prepared according to the manufacturer's instructions and applied to the pulp wound. The cavity is sealed as a temporary restoration. A final composite restoration is performed two weeks after pulp capping if pulp vitality is positive and there are no symptoms. Immediate and follow-up radiographs are taken to assess the tightness of the filling. The statistical analysis was performed using the "Microsoft Office Excel software" program (2019) for Windows. Figure 1 shows examples of the treated teeth.

## Results

Forty-three teeth of 37 patients were treated with direct and indirect pulp capping using Biodentine. Three patients did not return for follow-up, one at three months and two at six months, and were thus excluded from the study. This left 40 capped teeth in 34 patients that were followed up to six months. The demographic characteristics of the patients included 67.5% female and 32.5% male, with an age range of 15 to 53 years and a mean of 30.5 years with a standard deviation of 9.8. Of the 40 pulp cappings, 60% were maxillary and 40% mandibular, with 30% anterior, 35% premolars, and 35% molars (Table 2). 

**Table 2:** Descriptive results of different variables of the study

Studied variables	Total	Success rate
	N	N(%)
Gender		
Female	27	23 (85)
Male	13	9 (69)
Age group, years		
15 - 34	25	18(72)
35 - 55	15	14 (93)
Tooth location		
Maxilla	24	20 (83)
Mandible	16	12 (75)
Tooth type		
Anterior	12	11 (92)
Premolar	14	11 (79)
Molar	14	10 (71)
Caries treatment		
Initial	35	29 (83)
Secondary	5	3 (60)
Baume types		
Type I	26	21 (81)
Type II	14	11 (79)
Caries location		
Occlusal	8	7 (88)
Proximal	32	25 (78)
Type of capping		
Direct	23	17 (74)
Indirect	17	15 (88)

The thermal pulp testing revealed exaggerated pain on six teeth at one month and on two others at three months, as well as delayed pain on two teeth at six months. Three teeth that showed exaggerated pain at one month, without clinical signs, became normal in sensitivity testing at both the three- and six-month mark and were thus considered successful. The results according to socio-demographic characteristics, tooth-related parameters, and type of styling are summarized in Table 2.

The success of the pulp capping was determined through postoperative clinical and radiographic evaluations at one month, three months, and six months. Successful capping of pulps was determined when patients reported no pain or discomfort, responded positively to the sensitivity test without increased thermal sensitivity or pain on percussion, and with no radiological signs of periapical pathology. The overall success rate was 90% at one month, 85% at three months, and 80% at six months (Table 3).

**Table 3:** Results after six months of follow-up

Pulp capping	Number	Percentage
	N	%
Success	32	80
Failure	8	20
Total	40	100

## Discussion

This clinical study assessed the pulp reaction to direct and indirect Biodentine pulp capping on 40 decayed teeth performed at the Department of Conservative Dentistry and Endodontics in Casablanca. Indirect capping is recommended as an alternative to direct capping in very deep caries to avoid pulp exposure. Indeed, pulp capping has included cases of accidental exposure and deep cavities where the pulp is visible by transparency after caries curettage.

On the other hand, for cases of indirect pulp capping in deep cavities where the residual dentin thickness is less than 0.5mm, the number and size of the "open" tubulin are such that communication with the pulp parenchyma is comparable to that of a true exposure [15].

The senior responsible for the study performed caries curettage with a dental dam, capping procedure, follow-up and data collection, and interpretation of the results. The pulp capping was completed during the same session of pulp exposure or during the curettage of deep caries, which allowed for a more efficient and confident management of the cases. The skill of the operator has been noted to be an influential factor in the success of pulp capping procedures. 

The clinical indication for pulp vitality therapy is a subject of debate in endodontic literature. Several variables such as patient age, tooth type, bleeding time, number and size of pulp exposure, capping material, pathological state of the remaining pulp tissue, clinical diagnosis, and the number and experience of treatment providers have been suggested to influence the indication and outcome of pulp capping procedures [16-19]. In a study conducted in this regard, 30% of the capping performed involved anterior teeth and 70% involved posterior teeth. It was found that the location of the tooth and the type of tooth had no influence on the success or failure of direct pulp capping, with the success rate being 91.66% for anterior teeth, 78.57% for premolars, and 71.42% for molars [12,20,21].

Pulp diagnosis

In our study, we used subjective information reported by the patients, as well as clinical and radiographic examinations to determine the preoperative and postoperative pulp diagnosis. However, a clinical evaluation does not allow an exact diagnosis of the pulp status, as it can only be obtained by means of histological examination [22]. Ricucci et al. have shown that the clinical diagnosis of reversible pulpitis correlates with the histological diagnosis of reversible pulpitis in 96% of cases, and in the case of irreversible pulpitis, the clinical diagnosis and the histological diagnosis correlate in 85% of cases [23]. This means that in one out of seven cases, there is a risk of endodontic treatment of a tooth with reversible pulpitis. Since the capped teeth were not intended for extraction, a histological analysis could not be applied in a clinical study such as ours. We used the cold test to test pulp sensitivity, as it is the simplest and most widely used method for assessing pulpal sensitivity in our population. However, this test only gives an indication of pulp vitality, as the “vitality” of a tooth is defined by sufficient blood flow, while “sensitivity” is defined by the reactive nerve support of the pulp tissue. The blood supply is of greater importance in maintaining the vitality of pulp tissue, but it cannot be determined clinically or on a radiograph.

In this study, teeth with deep caries or reversible pulpitis were analyzed, and the success rate for direct pulp capping was 81% and 79%, respectively. This is in line with the findings of a systematic review and meta-analysis conducted by Paula Ruiz-González et al., which concluded that direct pulp capping is an effective technique with success rates of 80% or higher, even when preoperative diagnosis is compromised [24]. Therefore, direct pulp capping should be considered a viable therapeutic option for the treatment of irreversible pulpitis.

Pulp capping material

Since the introduction of calcium silicate materials, calcium hydroxide has been used less and less in pulp vitality therapies due to its limited adhesion to the tooth walls, instability over time, potential for tunnel defects in the dentin bridge, and unpredictable success rates [25]. 

A systematic review and meta-analysis conducted in 2020 by Cushley et al. revealed that the success rate of pulp capping using calcium hydroxide was 74% at six months, 65% at one year, 59% at two or three years, and 56% at four or five years [26]. This success rate is lower than that of direct capping with MTA (91%, 86%, 84%, and 81% respectively) and Biodentine (96%, 86%, and 86% respectively). No significant difference was noted between MTA and Biodentine [26,27]. 

The Biodentine material offers several advantages over calcium hydroxide; it has a shorter processing time, is mechanically stronger and less soluble, and provides a tighter seal. This qualifies it to avoid three major disadvantages of calcium hydroxide: material resorption, mechanical instability, and failure to prevent the resulting micro-percolation. In addition, it is bioactive and stimulates progenitor cells or pulp stem cells and forms a reparative dentin-like matrix secreted by odontoblast-like differentiated cells. These pulp cells synthesize osteodentin and are the first wave of cells involved in the formation of the dentinal bridge during pulpal exposure [28]. However, in a long-term retrospective study using calcium hydroxide in direct capping, the success rate in the first 10 years of follow-up was 95% and 89% after 35 years of follow-up. The quality or presence of the coronal restoration was the main variable significantly affecting the treatment outcome [29]. 

MTA and Biodentine are commonly used for direct pulp capping, a technique used to protect the dental pulp from further damage. MTA is known for its ability to form a hard mineralized bridge that prevents inflammation of the pulp, with similar clinical rates of efficacy as Biodentine [30]. Biodentine, however, is advantageous because it can be used for direct and indirect pulp capping, as well as for temporary filling of cavities up to six months [12,31]. Furthermore, Biodentine is easier to handle and requires a shorter setting time than MTA and can be used simultaneously as a pulp protection and temporary filling [32,33].

Acid etching of Biodentine has been shown to cause structural and chemical changes to the surface of the material, weakening the adhesion of composites to the freshly mixed Biodentine surface [34]. However, delaying the placement of the final composite restoration for at least two weeks after the placement of Biodentine can increase the bond strength due to resistance to shrinkage of the composite resins [35]. On the other hand, resin-modified glass ionomer cement has been found to have a higher shear bond strength value in resin composite restorations compared to the three calcium silicate liners MTA, Biodentine, and TheraCal LC, potentially making it the best material for use in this situation [36].

Follow-up period 

Our study was conducted over a six-month period, providing a reliable prognosis of the pulp vitality. According to Matsuo et al., pulp exposures have a success rate of 81.8% at three months and 81.3% at 18 months, suggesting that the majority of failures occur within the first three months following treatment [21]. Therefore, our six-month study duration was suitable for providing an accurate assessment of treatment outcomes. However, follow-up should continue beyond this period as failure may occur several months or years after treatment. We achieved a success rate of 85% at three months and 80% at six months for Biodentine pulp capping. Two additional cases of failure were recorded after six months. A longer-term follow-up of our cases is necessary to establish an accurate evaluation of clinical success. It is recommended to conduct follow-ups at 3-4 weeks, three months, six months, and 12 months for radiographic control and once a year thereafter [37]. Success is measured by the absence of pain or other symptoms, the absence of signs of internal root resorption, apical periodontitis, and the maintenance of pulp vitality for at least one year.

The ESE (European Society of Endodontics) in 2019 recommended follow-up periods for pulp vitality therapeutics by clinical evaluation at six months and clinical/radiographic evaluation at 12 months and at annual intervals (if necessary) for four years thereafter [16]. Ideally, the tooth should respond positively to pulp sensitivity testing (thermal +/- electrical) without persistent or exaggerated response [18].

The current study has some limitations. To ensure hemostasis, we used sodium hypochlorite and saline for rinsing. Although all solutions are recommended to control hemorrhage, sodium hypochlorite has been shown to inhibit the differentiation of odontoblasts from dental pulp stem cells [38]. In addition, the sample size can be increased and the follow-up period to evaluate the long-term success rate.

Limitations 

We were unable to include very young patients in our study because the Department of Conservative Dentistry and Endodontics receives generally elderly patients. This allowed us to compare the success rate with that of young patients reported in the literature. Indeed, younger patients' pulp is thought to have a better capacity for healing than older patients' pulp, which is more fibrous and less vascularized, and whose reparative potential is reduced with degenerative changes [12,39]. Also, the lack of blinding in the evaluation process may introduce bias in the assessment of pulp sensitivity and radiographic analyses. This series of clinical cases allowed us to increase the sample size and take these limitations into consideration. 

## Conclusions

Biodentine's moderate cost and short setting time make it an ideal material for both indirect and direct pulp capping in clinical practice. Its easy-to-use nature also makes it a great option for students, allowing them to gain the experience they need to become skilled dentists. This study presents a series of clinical cases to demonstrate the effectiveness of Biodentine for pulp capping.

## References

[1] (2006). Quality guidelines for endodontic treatment: consensus report of the European Society of Endodontology. Int Endod J.

[2] Schwendicke F, Brouwer F, Schwendicke A, Paris S (2016). Different materials for direct pulp capping: systematic review and meta-analysis and trial sequential analysis. Clin Oral Investig.

[3] Hilton TJ (2009). Keys to clinical success with pulp capping: a review of the literature. Oper Dent.

[4] Baume LJ, Holz J (1981). Long term clinical assessment of direct pulp capping. Int Dent J.

[5] Prosser HJ, Groffman DM, Wilson AD (1982). The effect of composition on the erosion properties of calcium hydroxide cements. J Dent Res.

[6] Cox CF, Sübay RK, Ostro E, Suzuki S, Suzuki SH. (1996). Tunnel defects in dentin bridges: their formation following direct pulp capping. Oper Dent.

[7] Kitasako Y, Ikeda M, Tagami J (2008). Pulpal responses to bacterial contamination following dentin bridging beneath hard-setting calcium hydroxide and self-etching adhesive resin system. Dent Traumatol.

[8] Aguilar P, Linsuwanont P (2011). Vital pulp therapy in vital permanent teeth with cariously exposed pulp: a systematic review. J Endod.

[9] Brizuela C, Ormeño A, Cabrera C, Cabezas R, Silva CI, Ramírez V, Mercade M (2017). Direct pulp capping with calcium hydroxide, mineral trioxide aggregate, and Biodentine in permanent young teeth with caries: a randomized clinical trial. J Endod.

[10] Malkondu Ö, Karapinar Kazandağ M, Kazazoğlu E (2014). A review on biodentine, a contemporary dentine replacement and repair material. Biomed Res Int.

[11] Rajasekharan S, Martens LC, Cauwels RG, Anthonappa RP (2018). Biodentine™ material characteristics and clinical applications: a 3 year literature review and update. Eur Arch Paediatr Dent.

[12] Dammaschke T, Leidinger J, Schäfer E (2010). Long-term evaluation of direct pulp capping--treatment outcomes over an average period of 6.1 years. Clin Oral Investig.

[13] Murray PE, Windsor LJ, Smyth TW, Hafez AA, Cox CF (2002). Analysis of pulpal reactions to restorative procedures, materials, pulp capping, and future therapies. Crit Rev Oral Biol Med.

[14] Silva AF, Tarquinio SB, Demarco FF, Piva E, Rivero ER (2006). The influence of haemostatic agents on healing of healthy human dental pulp tissue capped with calcium hydroxide. Int Endod J.

[15] Smith AJ, Murray PE, Lumley PJ (2002). Preserving the vital pulp in operative dentistry: I. A biological approach. Dent Update.

[16] Duncan HF, Galler KM, Tomson PL (2019). European Society of Endodontology position statement: management of deep caries and the exposed pulp. Int Endod J.

[17] (2021). AAE Position Statement on Vital Pulp Therapy. J Endod.

[18] Zhu C, Ju B, Ni R (2015). Clinical outcome of direct pulp capping with MTA or calcium hydroxide: a systematic review and meta-analysis. Int J Clin Exp Med.

[19] Cho SY, Seo DG, Lee SJ, Lee J, Lee SJ, Jung IY (2013). Prognostic factors for clinical outcomes according to time after direct pulp capping. J Endod.

[20] Al-Hiyasat AS, Barrieshi-Nusair KM, Al-Omari MA (2006). The radiographic outcomes of direct pulp-capping procedures performed by dental students: a retrospective study. J Am Dent Assoc.

[21] Matsuo T, Nakanishi T, Shimizu H, Ebisu S (1996). A clinical study of direct pulp capping applied to carious-exposed pulps. J Endod.

[22] Hyman JJ, Cohen ME (1984). The predictive value of endodontic diagnostic tests. Oral Surg Oral Med Oral Pathol.

[23] Ricucci D, Loghin S, Siqueira JF Jr (2014). Correlation between clinical and histologic pulp diagnoses. J Endod.

[24] Ruiz-González P, Cabanillas-Balsera D, Saúco-Márquez JJ, Segura-Egea JJ (2022). Outcome of direct pulp capping in teeth diagnosed as irreversible pulpitis: systematic review and meta-analysis. J Clin Exp Dent.

[25] Cox CF, Sübay RK, Ostro E, Suzuki S, Suzuki SH (1996). Tunnel defects in dentin bridges: their formation following direct pulp capping. Oper Dent.

[26] Cushley S, Duncan HF, Lappin MJ, Chua P, Elamin AD, Clarke M, El-Karim IA (2021). Efficacy of direct pulp capping for management of cariously exposed pulps in permanent teeth: a systematic review and meta-analysis. Int Endod J.

[27] Alsubait S, Aljarbou F (2021). Biodentine or mineral trioxide aggregate as direct pulp capping material in mature permanent teeth with carious exposure? A systematic review and meta-analysis. Oper Dent.

[28] Laurent P, Camps J, About I (2012). Biodentine(TM) induces TGF-β1 release from human pulp cells and early dental pulp mineralization. Int Endod J.

[29] Ricucci D, Rôças IN, Alves FR, Cabello PH, Siqueira JF Jr (2023). Outcome of direct pulp capping using calcium hydroxide: a long-term retrospective study. J Endod.

[30] Nowicka A, Lipski M, Parafiniuk M (2013). Response of human dental pulp capped with biodentine and mineral trioxide aggregate. J Endod.

[31] Laurent P, Camps J, De Méo M, Déjou J, About I (2008). Induction of specific cell responses to a Ca(3)SiO(5)-based posterior restorative material. Dent Mater.

[32] Koubi G, Colon P, Franquin JC, Hartmann A, Richard G, Faure MO, Lambert G (2013). Clinical evaluation of the performance and safety of a new dentine substitute, Biodentine, in the restoration of posterior teeth - a prospective study. Clin Oral Investig.

[33] Bachoo IK, Seymour D, Brunton P (2013). A biocompatible and bioactive replacement for dentine: is this a reality? The properties and uses of a novel calcium-based cement. Br Dent J.

[34] Camilleri J (2013). Investigation of Biodentine as dentine replacement material. J Dent.

[35] Hashem DF, Foxton R, Manoharan A, Watson TF, Banerjee A (2014). The physical characteristics of resin composite-calcium silicate interface as part of a layered/laminate adhesive restoration. Dent Mater.

[36] Manoj A, Kavitha R, Karuveettil V, Singh VP, Haridas K, Venugopal K (2022). Comparative evaluation of shear bond strength of calcium silicate-based liners to resin-modified glass ionomer cement in resin composite restorations - a systematic review and meta-analysis. Evid Based Dent.

[37] Edward J, Swift JR, Martin T, Ritter AV (2003). Vital pulp therapy for the mature tooth - can it work?. Endod Topics.

[38] Kawashima N (2012). Characterisation of dental pulp stem cells: a new horizon for tissue regeneration?. Arch Oral Biol.

[39] Horsted P, Sandergaard B, Thylstrup A, El Attar K, Fejerskov O (1985). A retrospective study of direct pulp capping with calcium hydroxide compounds. Endod Dent Traumatol.

